# Impaired Spatio-Temporal Predictive Motor Timing Associated with Spinocerebellar Ataxia Type 6

**DOI:** 10.1371/journal.pone.0162042

**Published:** 2016-08-29

**Authors:** Robin Broersen, Yoshiyuki Onuki, Abdel R. Abdelgabar, Cullen B. Owens, Samuel Picard, Jessica Willems, Henk-Jan Boele, Valeria Gazzola, Ysbrand D. Van der Werf, Chris I. De Zeeuw

**Affiliations:** 1 Netherlands Institute for Neuroscience, Royal Dutch Academy of Arts & Sciences, Amsterdam, The Netherlands; 2 Department of Neuroscience, Erasmus Medical Center, Rotterdam, The Netherlands; 3 Department of Psychology, University of Amsterdam, Amsterdam, The Netherlands; 4 Department of Anatomy and Neurosciences, VU University Medical Center, Amsterdam, The Netherlands; Duke University, UNITED STATES

## Abstract

Many daily life activities demand precise integration of spatial and temporal information of sensory inputs followed by appropriate motor actions. This type of integration is carried out in part by the cerebellum, which has been postulated to play a central role in learning and timing of movements. Cerebellar damage due to atrophy or lesions may compromise forward-model processing, in which both spatial and temporal cues are used to achieve prediction for future motor states. In the present study we sought to further investigate the cerebellar contribution to predictive and reactive motor timing, as well as to learning of sequential order and temporal intervals in these tasks. We tested patients with spinocerebellar ataxia type 6 (SCA6) and healthy controls for two related motor tasks; one requiring spatio-temporal prediction of dynamic visual stimuli and another one requiring reactive timing only. We found that healthy controls established spatio-temporal prediction in their responses with high temporal precision, which was absent in the cerebellar patients. SCA6 patients showed lower predictive motor timing, coinciding with a reduced number of correct responses during the ‘anticipatory’ period on the task. Moreover, on the task utilizing reactive motor timing functions, control participants showed both sequence order and temporal interval learning, whereas patients only showed sequence order learning. These results suggest that SCA6 affects predictive motor timing and temporal interval learning. Our results support and highlight cerebellar contribution to timing and argue for cerebellar engagement during spatio-temporal prediction of upcoming events.

## Introduction

Many daily life activities demanding immediate timed motor responses require integration of spatial and temporal information at the millisecond range (*e*.*g*. hitting a ball when playing tennis). Our ability to continuously create predictions based on spatial and temporal cues (‘spatio-temporal prediction’) aids us to make timed, coordinated movements and perceptual judgments in relation to changes in our environment. Using functional magnetic resonance imaging (fMRI), we have shown that co-activation of cerebellum and hippocampus occurred during spatio-temporal prediction when healthy participants were asked to make precisely timed finger movements based on moving visual cues, whereas no co-activation occurred during reactive timing or motor imagery tasks [1]. Interestingly, the participants tended to press buttons slightly prior to the optimal timing of visual cues as learning progressed, indicating anticipation in their responses. Those findings suggest that the cerebellum and hippocampus cooperate to plan ahead and execute precise spatial and temporal motor responses. The exact cerebellar contribution to temporal aspects in this task however, remains uncertain. A previous study has shown that patients with spinocerebellar ataxia (SCA) are impaired at intercepting a moving target by performing a timed button press to launch another moving object for collision, a task also requiring spatio-temporal prediction [2]. Only patients with disorders that affect the cerebellum such as SCA type 6 and 8 and head essential tremor (ET), scored significantly worse on this predictive timing task when compared to those with other neurological disorders, such as Parkinson’s disease [3]. During the same task, higher blood oxygenation level-dependent (BOLD) signals in several cerebellar regions as well as thalamus and multiple cortical areas were associated with successful performance. In fact, the activity change during successful trials in several cerebellar regions was found to be higher in controls compared to SCA patients, indicating a role for the cerebellum in spatio-temporal prediction during target interception [4]. In a velocity judgement task that required purely perceptual spatio-temporal prediction, the posterior cerebellar lobule VII crus I was engaged when both temporal and spatial cues, but not when only spatial cues were used. Psychophysiological interactions (PPI) analysis further revealed that connectivity between the posterior cerebellum and several other upstream areas in the brain increased during spatio-temporal prediction in the perceptual domain [5]. This suggests that the cerebellum is important when integration of temporal information is required to complete the task goal.

Pioneering work to determine cerebellar contribution to timing processes has been done by Ivry and colleagues [6–9]. Their work has received support from multiple studies in which timing elements of tasks were used explicitly or implicitly, depending on whether participants were required to use an overt estimation of time or to use time as a by-product to reach the task goal, respectively [10]. Deficits at both explicit timing tasks (*e*.*g*. temporal discrimination, temporal reproduction and synchronized repetitive finger-tapping tasks) and implicit timing tasks (*e*.*g*. spatio-temporal trajectory prediction and serial prediction tasks) have been found in patients with cerebellar disorders [2,3,6,11–16]. These findings complement evidence from neuroimaging studies showing cerebellar activation associated with temporal processing [4,17–19]. Disrupted cerebellar activity would likely be accountable for the observed deficits in these patients. Tasks employing repetitive transcranial magnetic stimulation (rTMS) furthermore show that temporal processing can be disrupted by interfering with cerebellar activity [20,21]. Together, these studies strongly implicate the cerebellum as part of a wider neuronal network involved in timing functions [9].

In the present study, we investigated the role of the cerebellum in spatio-temporal prediction of moving visual stimuli. We tested patients with spinocerebellar ataxia type 6 (SCA6) and healthy age-matched controls on two related motor tasks: a predictive and a reactive motor timing task. This allowed us to study cerebellar contribution to predictive and reactive motor timing functions separately. We further employed an experimental design in which we randomized the sequence order of markers and temporal intervals between them so as to investigate cerebellar involvement in sequence order and temporal interval learning. We hypothesized that cerebellar patients would show a reduced temporal precision in responses, coinciding with reduced performance on the task requiring spatio-temporal prediction, but not on the reactive timing task. Furthermore we expected that cerebellar patients would be impaired at both sequence order and temporal interval learning. SCA6 patients form an adequate subject group to test these hypotheses, since the atrophy specifically affects the cerebellum in adults [22,23]. SCA6 is an autosomal dominant genetic disorder caused by a CAG repeat expansion in exon 47 of the CACNA1A gene, which encodes the α1A (Ca_v_2.1) subunit of neuronal P/Q-type voltage-gated calcium channel. This causes essentially pure cerebellar atrophy with a profound loss of Purkinje cells [24] and manifests with a range of symptoms including imbalance, dysarthria, gait ataxia, upper limb incoordination and tremor [25,26].

Our main finding is that SCA6 patients failed to establish spatio-temporal prediction in timing of their responses, reflecting a deficit in motor anticipation to upcoming events. Whereas healthy participants timed their button presses on average before the optimal timing with better temporal precision, this anticipation was absent in SCA6 patients. Performance of patients was reduced during the predictive timing task, but this deficit was limited to early correct responses that were made during the anticipatory period of the task. On the reactive timing task, randomization of sequence order of stimuli and temporal intervals between them resulted in reduced performance of the healthy controls. In contrast, randomization of temporal intervals did not further reduce performance in SCA6 patients. In summary, our findings suggest that cerebellar dysfunction may impair spatio-temporal prediction and prevent temporal interval learning to occur. These findings lend support to leading views on cerebellar involvement in timing functions.

## Subjects and Methods

### Participants

We tested 19 patients with spinocerebellar ataxia type 6 (SCA6) (mean age 61.8 ± 7.2, standard deviation (SD), range 49–80) and 14 healthy control participants (mean age 64.5 ± 4.7, SD, range 58–74) for this experiment. We attempted to achieve homogeneity across participants by using several exclusion criteria, including suffering from fatigue, neuropsychological disorders or expertise with musical instruments, since our task requires skills often used in, for example, piano and guitar playing. We excluded 7 patients from our analysis based on the occurrence of severe tremors (unilateral or bilateral) and/or excessive fatigue and 2 control participants based on technical issues of the behavioral task during the experiment. We included the remaining participants: 12 SCA6 patients (3 males; 2 left-handed) and 12 healthy control participants (7 males; 2 left-handed) in our data analysis. The average age of these SCA6 patients was 60.3 ± 6.4, SD (range 49–67) and 64.8 ± 4.8, SD (range 58–74) of control participants [*t*_22_ = 1.985, *P* = 0.06, two-sample *t*-test]. We did not detect significant correlations between age and performance on the predictive timing task (*r* = 0.139, *P* = 0.517, Pearson correlation) or performance on the reactive timing task (*r* = -0.213, *P* = 0.318, Pearson correlation). All participants met the criteria of normal or corrected-to-normal vision, no excessive computer gaming or playing musical instruments for more than five hours a week and no history of other neuropsychological or motor disorders at the time of measurements or in the past. Participants were instructed prior to measurements and a written informed consent was obtained from all participants. A neurological exam was performed by a certified neurologist prior to the experiment to obtain a measurement of severity of cerebellar ataxia, according to the guidelines of the Scale for the Assessment and Rating of Ataxia (SARA) [27]. The average SARA scores in SCA6 patients was 9.5 ± 5.2, SD on a scale of 0–40. This score is the average of scores on eight different subtasks, comprising an assessment of gait, stance, sitting, speech, finger and hand movements, and leg coordination. A score of 0 indicates that no neurological manifestations could be observed, whereas a score of 40 reflects the occurrence of severe neurological manifestations [27]. This study was approved by the medical ethical committee of Erasmus MC, Rotterdam (MEC-2013-095).

### Behavioral task

**Fig 1:**
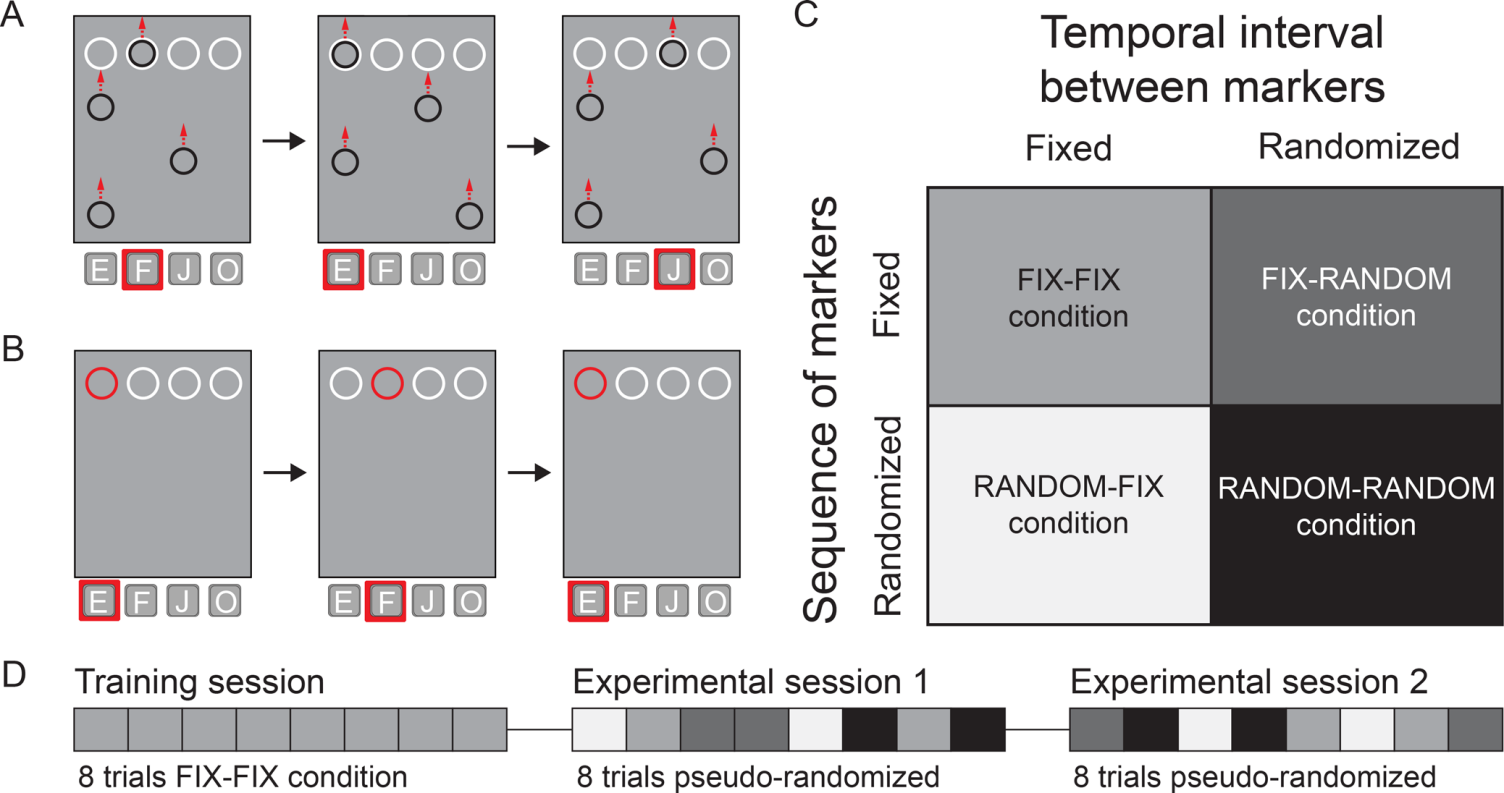
Behavioral task and experimental conditions. (A) During the predictive timing task black markers move from the bottom towards the four white target markers at the top of the screen. Participants are instructed to press the corresponding key at the moment that the black moving marker fully overlaps with the target marker (red square highlights the corresponding key). Target markers (from left to right) correspond to the ‘e’, ‘f’, ‘j’ and ‘o’ keys of a keyboard. (B) In the reactive timing task the white target markers change color to red for 200ms and participants are instructed to press the corresponding key as fast as possible. (C) Trials have one of four possible task conditions based on the sequence of and temporal interval between markers (fixed or randomized). (D) Both in the reactive timing task and predictive timing task, participants first complete a training session containing 8 trials of the FIX-FIX condition (*i*.*e*. fixed sequence and fixed interval), followed by two experimental sessions in which 2 trials of each condition are presented in a pseudo-randomized order.

All participants completed the entire experiment, which included two modified versions of the Serial Interception Sequence Learning task [1,28]: a predictive timing task and a reactive timing task. In both tasks (Fig 1A and 1B), four empty white circles were placed on top of a gray rectangular background screen. Each of the white circles corresponded to four keys on a standard Qwerty-keyboard, which in turn were associated to a pre-determined finger: the ‘e’ key corresponded to the left middle finger, the ‘f’ key to the left index, the ‘j’ key to the right index and the ‘o’ key to the right middle. In the predictive timing task, participants were instructed to press the corresponding button on the keyboard at the moment upward moving black circles (moving markers) completely overlapped with the corresponding static white circles (target markers) at the top of the screen (Fig 1A). Only one moving marker could overlap with a target marker at a given moment. The diameter of the moving markers was 20 pixels smaller than that of the target marker to fit completely inside the target markers and not to interfere with the moment of the complete overlap between markers as observed by the participant. Moving markers scrolled from the bottom of the screen to the top at a constant velocity (2.78 milliseconds per pixel). In the reactive timing task, participants were instructed to press the corresponding button on the keyboard as quickly as possible when the white target marker changed (flashed) to red for 200 milliseconds (Fig 1B). Only one target marker could flash at a given moment. Between each trial, a short task instruction appeared on the screen for 3 seconds: *‘Press on the corresponding button with your finger as soon as the circles entirely overlap’* and *‘Press on the corresponding button with your finger as soon as a circle flashes’* (translated from Dutch), during the predictive and reactive task, respectively. The duration of this task instruction formed the only inter-trial interval time. Between each session, which lasted on average 7.9 ± 0.43 (SD) minutes participants took a short break (1–2 minutes) to reduce physical and mental fatigue. No feedback to indicate correct responses was given during the task. Both tasks were programmed in MATLAB R2010a (MathWorks, Massachusetts, USA) using Psychtoolbox-3 [29,30].

### Task conditions

We studied the role of the cerebellum in sequence and temporal interval learning by employing in both tasks a 2-by-2 factorial task design with two factors: the sequential order of stimuli and temporal interval between them. The sequences of moving markers (or marker flashes) and the temporal intervals between stimuli were either according to a pre-defined order (FIX) or shuffled based on the pre-defined order (RANDOM) (Fig 1C). The combination of these resulted in four task conditions: FIX-FIX (*i*.*e*. fixed sequence and fixed intervals), FIX-RANDOM, RANDOM-FIX and RANDOM-RANDOM. For each condition, a sequence of 12 circles was presented 4 consecutive times, which resulted in 48 moving or flashing markers that were presented during each trial. In FIX-FIX condition of the predictive timing task, the sequence of moving markers followed the same order 4 consecutive times: F_1386ms_-E_718ms_-J_852ms_-E_852ms_-O_852ms_-E_702ms_-J_969ms_-F_952ms_-J_1887ms_-O_668ms_-F_1420ms_-O_668ms_ (E: left-most trajectory, F: second trajectory, J: third trajectory, O: right-most trajectory, subscripted rates: temporal differences between moving markers in milliseconds). In FIX-FIX condition of the reactive timing task, the sequence of the marker flashes was E_707ms_-F_1345ms_-E_1078ms_-O_849ms_-J_1200ms_-F_703ms_-O_1490ms_-E_844ms_-J_1824ms_-O_1180ms_-F_811ms_-J_1075ms_. The average temporal interval between appearances was 1001 milliseconds (range 668–1887 milliseconds) in the predictive timing task and 1092 milliseconds (range 703–1824 milliseconds) in the reactive timing task.

### Experimental protocol

All experiments were conducted in the participants’ homes. A 13 or 15 inch computer screen was used to present the visual stimuli with a resolution of 1280 x 800 pixels (13 inch screen: all SCA6 patients and 4 out of 12 control participants). To rule out that screen size had an effect on performance, we compared performance on both tasks of controls tested using a 13 inch screen (N = 4) versus controls tested using a 15 inch screen (N = 8). We did not find significant differences between these controls on both the predictive timing task [*t*_*10*_ = 0.981, *P* = 0.35, two-sample *t*-test] and on the reactive timing task [*t*_*10*_ = 0.869, *P* = 0.405]. Participants were instructed prior to the experiment by an information letter mailed in advance. The tasks were also verbally explained to the participants right before starting the tasks. A summary of the instructions was finally displayed on the screen before the task began. When participants confirmed they fully understood the tasks, we started the experiment. Each task consisted of one training session and two experimental sessions. During the training session, eight trials with the FIX-FIX condition were presented. In each experimental session, each task condition was presented twice in a pseudo-randomized order using randomization algorithms in MATLAB (Fig 1D). All SCA6 patients and 5 out of 12 control participants started with the predictive timing task, followed by the reactive timing task as part of an experimental sequence. The remaining control participants (N = 7) first started with the reactive timing task, directly followed by the predictive timing task. To rule out that the order of tasks had an effect on performance, we compared performance on both tasks of controls tested first on the predictive task (N = 5) versus controls tested first on the reactive task (N = 7). We did not find significant differences between these controls on both the predictive timing task [*t*_*10*_ = 0.157, *P* = 0.878, two-sample *t*-test] and on the reactive timing task [*t*_*10*_ = 0.727, *P* = 0.484, two-sample *t*-test].

### Analysis of behavioral data

Classification of correct responses depended on two factors, pressing the corresponding button and pressing the button within a given time window. To maximize the sensitivity of analyzing responses within a set time window, we calculated performance as the area under the curve of the correct ratio (AUCCR). The correct ratio was defined as the number of corresponding (correct) button presses within a time window, divided by the total number of markers within a trial. The minimum temporal interval between markers was 668 milliseconds in the predictive timing task and 703 milliseconds in the reactive timing task. Based on these minimum times, we used a time window between -334ms to +334ms relative to perfect marker overlap in the predictive timing task and 0 to 703ms after marker flash in the reactive timing task throughout our analysis. To assess spatio-temporal prediction in the predictive timing task, we calculated the AUCCR before marker overlap (-334ms to < 0ms) representing early correct responses and after marker overlap (> 0ms to +334ms), representing late correct responses. We ascertained normality of data distributions using the Shapiro-Wilk test. Unless indicated otherwise, we used one-way repeated measures analyses of variance (ANOVA) with trial number (for training sessions) or trial condition (for experimental sessions) as within-subject variable and group as between-subject variable to test AUCCR on both reactive and predictive tasks. We used the Mauchly’s test to test the assumption of sphericity and in case this assumption was violated, we applied the Greenhouse-Geisser correction. Levene’s test of equality of error variances was used to test whether the assumption of equal error variances was met. To analyze pooled response and reaction times, we used the non-parametric Wilcoxon rank sum test and Wilcoxon signed rank test with Bonferroni correction for multiple comparisons, since these distributions violated the assumption of normality. We compared variances of these distributions using the Brown-Forsythe test. Comparison of the first principal component (PC1) of the AUCCR of both tasks was done using independent-samples *t*-tests and a correlation analysis was performed using the Pearson correlation. These performance measures were also used in the methods section to test for effects of age, screen size and task order. Statistics were conducted in SPSS version 22 (IBM, New York, USA) and MATLAB R2011b (MathWorks, Massachusetts, USA). Results are reported as mean ± standard error of the mean (SEM), unless indicated otherwise.

## Results

### SCA6 patients show lower predictive motor timing performance, localized to early correct responses

**Fig 2:**
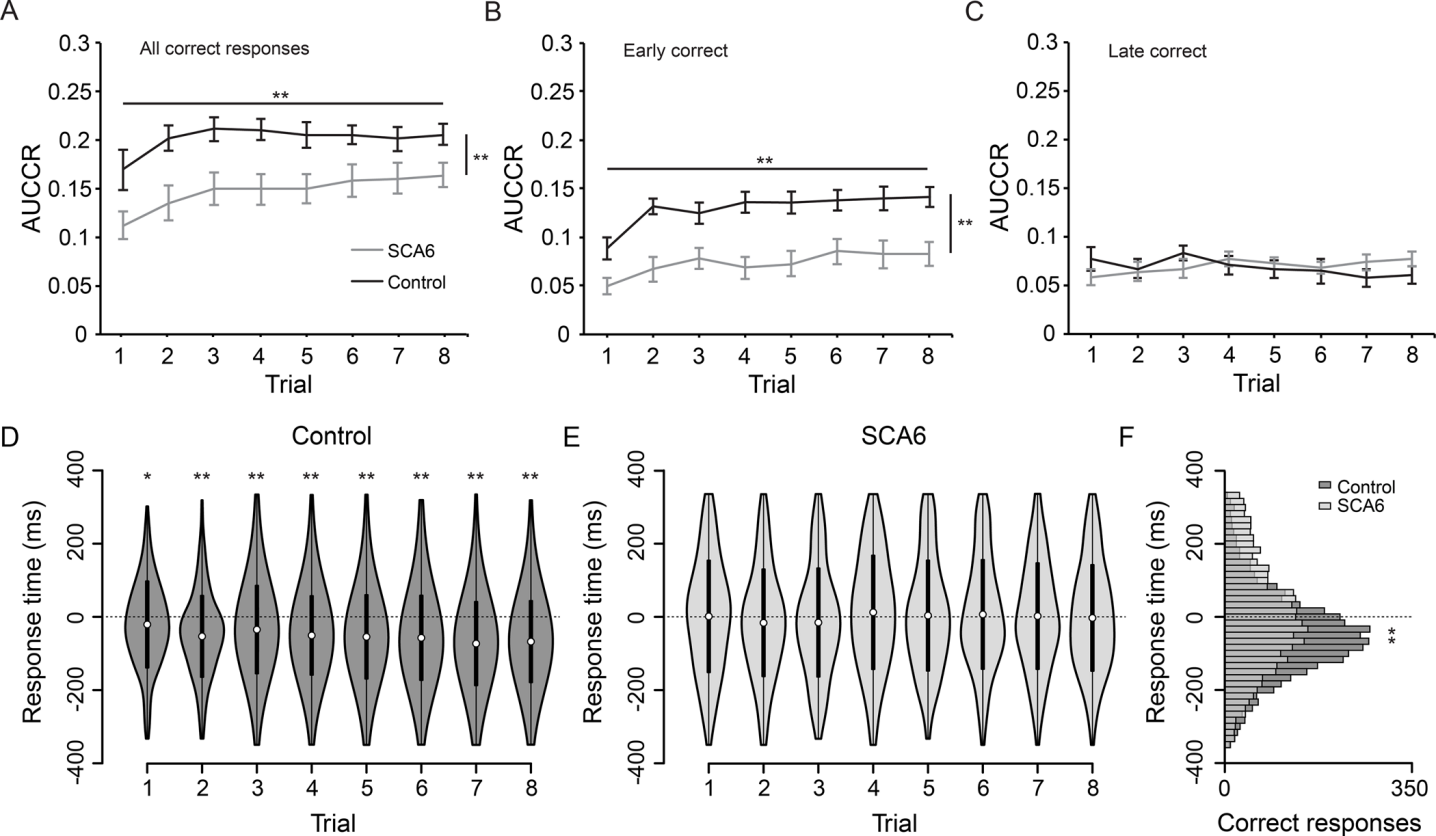
Results training session of predictive timing task. (A) Task performance showing all correct responses, expressed as area under the curve of the correct ratio (AUCCR) for both controls and SCA6 patients, showing a learning effect and a difference between groups. (B) AUCCR of early correct responses only, where the learning effect and group differences are present. (C) AUCCR of late correct responses only, where no learning effect or group differences are present. (D) Response times distributions of correct button presses of controls. Average response times are significantly lower than 0ms on all trials (*asterisks*). (E) Response times distributions of correct button presses of SCA6 patients. Average response times are not significantly different from 0ms on any of the trials. (F) Distribution of pooled response times for all training trials, showing that controls time their button press more precisely as observed by a smaller variance compared to SCA6 patients (*asterisks*). (A, B, C) Error bars represent SEM, ** indicates *P* < 0.01 for main effects of trials (*asterisks* horizontal line) and main effects of group (*asterisks* vertical line). (D, E) White dots represent the mean and black bars represent the SD. (F) Histogram shows 16.7ms bins. * indicates *P* < 0.05, ** indicates *P* < 0.01.

We used area under the curve of the correct ratio (AUCCR) to analyze performance of responses made within a temporal window around complete marker overlap (-334ms to +334ms; all correct responses). Over the course of the training session, both SCA6 patients and controls learned the sequence and timings of the markers (Fig 2A). Controls showed a significantly higher AUCCR compared to SCA6 patients during the training session, indicating that controls made more correct button presses within the assigned time window [main effect of trials: *F*_7,154_ = 7.44, *P* < 0.001; main effect of group: *F*_1,22_ = 10.23, *P* < 0.01; interaction trials x group: *F*_7,154_ = 0.87, *P* = 0.53]. To assess the effect of spatio-temporal prediction in our task, we segregated the data based on the time of button press. Early and late correct responses are corresponding button responses made before or after the moving marker completely overlapped with the target marker, respectively (Fig 2B and 2C). When analyzing early correct responses we found that controls showed a significantly higher AUCCR compared to SCA6 patients, although both groups showed a learning effect [main effect of trials: *F*_7,154_ = 10.67, *P* < 0.001; main effect of group: *F*_1,22_ = 15.56, *P* = 0.001; interaction trials x group: *F*_7,154_ = 1.25, *P* = 0.28]. In contrast, for late correct responses we found no significant differences in AUCCR between groups and no learning effect [main effect of trials: *F*_3.313, 72.883_ = 0.71, *P* = 0.56; main effect of group: *F*_1,22_ = 0.02, *P* = 0.89; interaction trials x group: *F*_3.313, 72.883_ = 2.4, *P* = 0.23, with Greenhouse-Geisser correction]. A third comparison including all factors revealed a significant interaction between groups and type of correct response (early versus late), confirming that the observed group difference was localized to early correct responses only [*F*_1, 22_ = 10.09, *P* = 0.004, with Greenhouse-Geisser correction; two-way repeated measures ANOVA]. In short, our data shows a performance difference between SCA6 patients and healthy controls during training on the predictive timing task, which is localized to (early) correct responses made during the anticipatory period of the paradigm.

### SCA6 patients show impaired anticipation in motor timing

To gain insight in the characteristics of motor timing in the predictive task, we visualized response time distributions of correct button presses of both controls and SCA6 patients (Fig 2D and 2E). Negative and positive response times correspond to button presses before and after perfect marker overlap, respectively. We pooled response times of correct button presses from all participants per group, since some SCA6 patients had only few correct responses per trial. This resulted in analyses based on a fixed template design. We calculated average response times for each trial of the training session: trial 1 [SCA6: 2.7 ± 154.8ms, control: -20.2 ± 120.3ms; mean ± SD], trial 2 [SCA6: -15.9 ± 148.3ms, control -51.9 ± 113.3ms], trial 3 [SCA6: -14.8 ± 150.3ms, control: -33.9 ± 122.2ms], trial 4 [SCA6: 12.1 ± 157.4ms, control: -49.1 ± 110ms], trial 5 [SCA6: 4.5 ± 152.8ms, control: -53.2 ± 116.6ms], trial 6 [SCA6: 6.9 ± 151.2ms, control: -55.9 ± 117.8ms], trial 7 [SCA6: 1.7 ± 147ms, control: -71.1 ± 116.3ms] and trial 8 [SCA6: -1.4 ± 147ms, control: -65.8 ± 113.5ms]. We found that response times of control participants were significantly lower than those of SCA6 participants on all trials, except on trial 1 and 3 [trial 1: mean difference (MD) = 22.9ms, Z = 2.42, *P* = 0.12; trial 2: MD = 36ms, Z = 4.26, *P* < 0.001; trial 3: MD = 19.1ms, Z = 2.53, *P =* 0.09; trial 4: MD = 61.2ms, Z = 7.0, *P* < 0.001; trial 5: MD = 57.7ms, Z = 6.22, *P* < 0.001; trial 6: MD = 62.8ms, Z = 7.24, *P* < 0.001; trial 7: MD = 72.8ms, Z = 8.37, *P* < 0.001; trial 8: MD = 64.4ms, Z = 7.31, *P* < 0.001; Wilcoxon rank sum test, with Bonferroni correction].

Previous findings have indicated that healthy participants establish spatio-temporal prediction during the training session of this predictive timing task [1], as indicated by average response times lower than 0ms. Indeed, this effect was also present in our control group, since we found that response times were significantly lower than 0ms on all trials of the training session [trial 1: Z = -2.84, *P* = 0.04; trial 2: Z = -9.51, *P* < 0.001; trial 3: Z = -6.5, *P* < 0.001; trial 4: Z = -9.46, *P* < 0.001; trial 5: Z = -9.85, *P* < 0.001; trial 6: Z = -10.16, *P* < 0.001; trial 7: Z = -12.13, *P* < 0.001; trial 8: Z = -11.65, *P* < 0.001; Wilcoxon signed rank test, with Bonferroni correction] (Fig 2D). In contrast, we did not find this effect in our SCA6 patient group, given that average response times of SCA6 patients were not different from 0ms on any of the training trials [trial 1: Z = -0.51, *P* = 1; trial 2: Z = -1.87, *P* = 1; trial 3: Z = -1.93, *P =* 1; trial 4: Z = -1.84, *P* = 1; trial 5: Z = -0.27, *P* = 1; trial 6: Z = -0.66, *P* = 1; trial 7: Z = -0.37, *P* = 1; trial 8: Z = -0.24, *P* = 1; Wilcoxon signed rank test, with Bonferroni correction] (Fig 2E). These findings indicate that spatio-temporal prediction is established during the training session in control participants, but not in SCA6 patients.

### SCA6 patients show reduced temporal precision in predictive motor timing

Since participants were instructed to time their button presses as closely as possible to perfect marker overlap, we investigated whether both groups were able to correctly time their button press. Therefore, we assessed whether there were differences in response time distributions between groups with variance as a measure of precision. A lower variance of response times would indicate a higher precision, whereas a higher variance would suggest a more temporally distributed response pattern, indicating a lower precision. To address this question, we compared the variance of pooled response time distributions of the training session between groups using the Brown-Forsythe test of variances, since this test is relatively robust and insensitive to deviations from normality [31]. We found that SCA6 patients showed a significantly higher variance of response times compared to controls [SCA6: 2.284x10^4^ ms^2^, control: 1.371x10^4^ ms^2^, *F*_1,5808.03_ = 239.98, *P* < 0.001] (Fig 2F). To verify that this difference was also present during the experimental sessions, we compared the variance of pooled response times of correct responses during the experimental sessions and we found that also here controls had a significantly higher variance [SCA6: 2.195x10^4^ ms^2^, control: 1.352x10^4^ ms^2^, *F*_1, 14580.35_ = 239.98, *P* < 0.001]. These results indicate that correct button presses were more temporally distributed in the SCA6 group, corresponding to a lower temporal precision of responses during both training and experimental sessions. Together, these findings suggest that healthy participants anticipate the upcoming event of a marker overlap with better temporal precision, whereas this anticipation of motor timing from the spatial and temporal cues is impaired in SCA6 patients.

### Sequential or temporal order randomization of markers does not affect predictive motor timing performance

**Fig 3:**
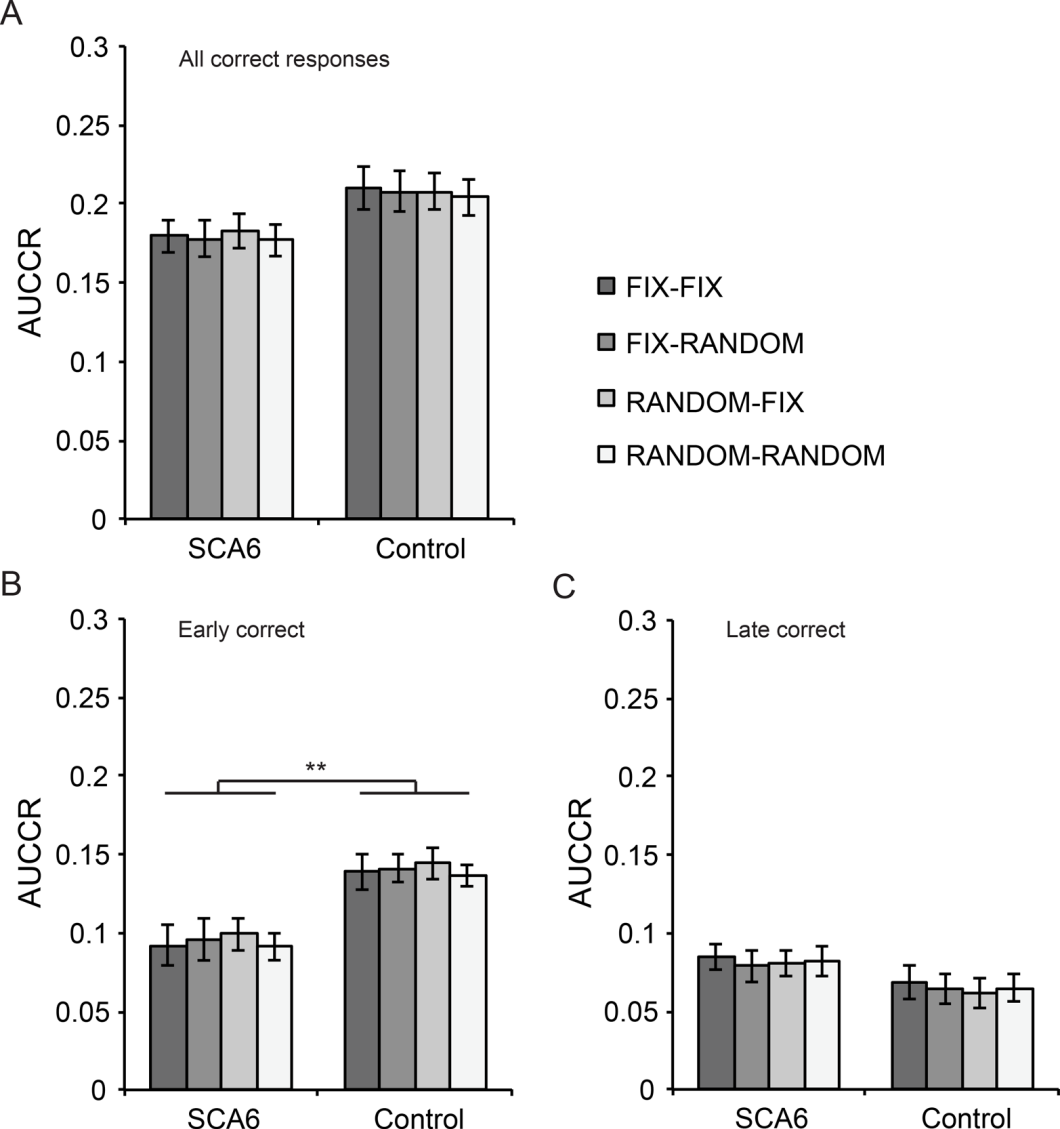
Results experimental sessions of predictive timing task. (A) Task performance on different trial conditions is visualized for both SCA6 patients and controls. No differences between groups or between conditions are observed in all correct responses. (B) AUCCR calculated for early correct responses shows a significant group difference between SCA6 patients and controls, but not between conditions. (C) AUCCR calculated for late correct responses does not show any differences between conditions or between groups. Error bars represent SEM, ** indicates *P* < 0.01.

We investigated sequential order and temporal interval learning by randomizing the order and temporal intervals of markers, resulting in four conditions that were presented during experimental sessions (Fig 1C and 1D). We calculated the AUCCR for both groups during different trial conditions: FIX-FIX condition [SCA6: 0.18 ± 0.01, control: 0.21 ± 0.013], FIX-RANDOM condition [SCA6: 0.178 ± 0.011, control: 0.208 ± 0.012], RANDOM-FIX condition [SCA6: 0.183 ± 0.011, control: 0.208 ± 0.011] and RANDOM-RANDOM condition [SCA6: 0.177 ± 0.01, control: 0.204 ± 0.011]. No significant differences in AUCCR between trial conditions or between groups were found, although the main effect of group approached the significance level and could be considered a trend (Fig 3A). Moreover, there was no interaction effect between conditions and groups [main effect of condition: *F*_3,66_ = 0.82, *P* = 0.49; main effect of group: *F*_1,22_ = 3.46, *P* = 0.08; interaction group x condition: *F*_*3*,*66*_ = 0.31, *P* = 0.82]. Therefore we could conclude from these data that randomizing sequences or temporal interval of markers did not lead to differences in performance or overall group differences when considering all correct responses.

Since we had found group differences in predictive motor timing for early correct responses during training (Fig 2B), we hypothesized that specifically these ‘anticipatory’ responses could be affected by randomization of sequence or temporal interval of markers. Therefore, we analyzed the data based on the timing of button presses and compared performance between conditions and groups (Fig 3B and 3C). We calculated the AUCCR of early correct responses on the FIX-FIX condition [SCA6: 0.091 ± 0.012, control: 0.139 ± 0.011], FIX-RANDOM condition [SCA6: 0.096 ± 0.012, control: 0.141 ± 0.009], RANDOM-FIX condition [SCA6: 0.099 ± 0.01, control: 0.144 ± 0.009], and RANDOM-RANDOM condition [SCA6: 0.091 ± 0.008, control: 0.137 ± 0.007] and we found that AUCCR differed significantly between groups. However, randomizing the sequence or temporal interval of markers did not lead to performance differences [main effect of condition: *F*_3,66_ = 1.94, *P* = 0.132; main effect of group: *F*_1,22_ = 11.95, *P* = 0.002; interaction group x condition: *F*_3,66_ = 0.38, *P* = 0.99] (Fig 3B). In addition, we investigated whether randomization had an effect on performance for late correct responses (Fig 3C). We calculated the AUCCR during the FIX-FIX condition [SCA6: 0.084 ± 0.007, control: 0.069 ± 0.01], FIX-RANDOM condition [SCA6: 0.079 ± 0.009, control: 0.064 ± 0.009], RANDOM-FIX condition [SCA6: 0.08 ± 0.007, control: 0.061 ± 0.009], and RANDOM-RANDOM condition [SCA6: 0.082 ± 0.009, control: 0.065 ± 0.008] and performed the same analysis. We found that AUCCR of late correct responses did not differ between groups or between conditions. Also, the data did not reveal an interaction effect [main effect of condition: *F*_3,66_ = 1.25, *P* = 0.30; main effect of group: *F*_1,22_ = 2.09, *P* = 0.16; interaction group x condition: *F*_3,66_ = 0.17, *P* = 0.92]. A third analysis including all factors confirmed that the significant group difference was localized to early correct responses only [interaction group x type of correct response (early versus late): *F*_1, 22_ = 10.01, *P* = 0.004; two-way repeated measures ANOVA]. Thus, although there were group differences in early correct responses, randomizing the order and temporal intervals of markers did not influence predictive motor timing performance regardless of the timing of responses.

### Lower predictive motor timing performance of SCA6 patients is limited to responses during the anticipatory period

To further compare performance of groups depending on the timing of response, but regardless of the trial condition, we conducted a principal component analysis (PCA) for dimension reduction. AUCCR scores for early correct responses between trial conditions were highly inter-correlated, at least *r* = 0.91 [*P <* 0.001, Pearson correlation]. Kaiser-Meyer-Olkin measure of sampling adequacy was 0.87, Bartlett’s test of sphericity was significant [χ^2^(6) = 142.67, *P* < 0.001] and all communalities were above 0.94, confirming that this data was suitable for PCA. We found that the first principal component (PC1) explained 95% of the variance and therefore we used this measure to represent performance of early correct responses. Next, we subjected AUCCR scores for late correct responses to PCA and consistently found a high inter-correlation between performance on different conditions [at least *r* = 0.85, *P <* 0.001, Pearson correlation]. Kaiser-Meyer-Olkin measure of sampling adequacy was 0.87, Bartlett’s test of sphericity was significant [χ^2^(6) = 101.11, *P* < 0.001] and all communalities were above 0.87. We found that the first principal component (PC1) explained 90.1% of the variance and therefore we used this measure to represent performance of late correct responses. We then used these measures to compare performance between groups, depending on the timing of response, regardless of the trial condition. We found a significant interaction effect between performance (PC1 of early vs late) and group [*F*_1, 22_ = 9.76, *P* = 0.005], although the main effect for group in this analysis did not reach the significance level [*F*_1,22_ = 3.65, *P* = 0.069]. *Post-hoc* tests confirmed that the group difference was localized to PC1 of early correct responses, but not to PC1 of late correct responses [early: *t*_22_ = 3.34, *P =* 0.006; late: *t*_22_ = -1.44, *P* = 0.328, two-sample *t*-test with Bonferroni correction]. In summary, although task performance was not influenced by randomization of sequences or temporal intervals, we observed a higher performance of early correct responses in controls only, which is in line with our results from the training session.

### SCA6 patients and controls perform comparably during reactive timing task training

**Fig 4:**
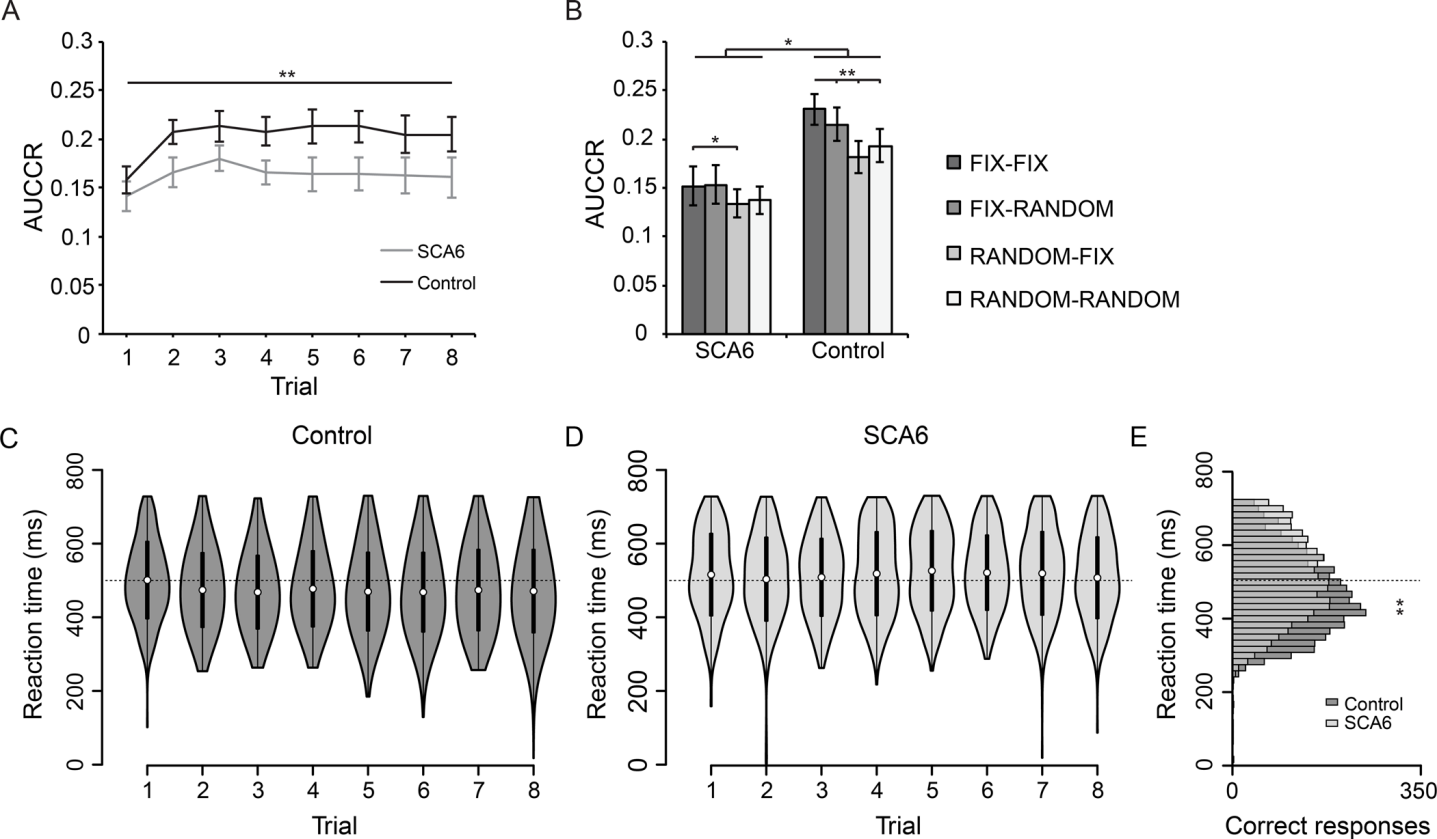
Results of reactive timing task. (A) Task performance expressed as AUCCR of correct responses after marker flash for both controls and SCA6 patients, showing that learning occurs over the course of the trials (*asterisks* horizontal line). (B) AUCCR calculated for different trial conditions during experimental sessions. The data shows group differences, as well as differences between all conditions with the FIX-FIX condition in the control group, but only between the RANDOM-FIX and the FIX-FIX condition in the SCA6 group. (C) Reaction time distributions of correct button presses during training for controls and (D) for SCA6 patients. (F) Distribution of pooled reaction times for all training trials. Variance of the reaction time distribution was smaller in controls (*asterisks*). (A, B) Error bars represent SEM, * indicates *P* < 0.05, ** indicates *P* < 0.01 for main effects of trials (*asterisks* horizontal line panel A) and main effects of condition and group (*asterisks* panel B). (D, E) Dotted line indicates 500ms for visual aid, white dots represent the mean and black bars represent the SD. (F) Histogram shows 16.7ms bins. ** indicates P < 0.01.

To investigate whether SCA6, in addition to predictive motor timing, also affected reactive motor timing functions, we tested all participants on a related reactive motor timing task where no prediction could be made based on visual cues. We calculated AUCCR of corresponding button presses made after marker flash (0-703ms) as performance measure during the training session, in which eight trials of the FIX-FIX condition were presented (Fig 1D). Since this task was purely reactive, we did not make a distinction between early and late responses. Both groups showed learning of the task, evidenced by an increase in AUCCR over the course of the training (Fig 4A). We did not observe a group difference, although P-value approximation to significance threshold suggested a trend. We also did not observe an interaction effect between groups and trials [main effect of trials: *F*_7,154_ = 9.88, *P* < 0.001; main effect of group: *F*_1,22_ = 3.47, *P* = 0.076; interaction trials x group: *F*_7,154_ = 1.41, *P* = 0.21]. In short, both groups increased their performance during the training, and overall there were no training differences between groups in this respect.

### SCA6 patients and controls show differences in reaction times and precision during reactive timing task training

We visualized reaction time distributions of the reactive timing task, showing development of reaction times of both groups over the training session (Fig 4C and 4D). We then calculated average reaction times of pooled correct responses on all trials of the training session: trial 1 [SCA6: 518.9 ± 113.8ms, control: 499.8 ± 107ms; mean ± SD], trial 2 [SCA6: 506.8 ± 115.8ms, control 477.4 ± 103.4ms], trial 3 [SCA6: 510 ± 107.6ms, control: 471.5 ± 102ms], trial 4 [SCA6: 521.6 ± 115.9ms, control: 480.4 ± 105.3ms], trial 5 [SCA6: 529.4 ± 111ms, control: 473.1 ± 108.8ms], trial 6 [SCA6: 524.2 ± 104ms, control: 471.7 ± 110.1ms], trial 7 [SCA6: 522.4 ± 115.5ms, control: 475.2 ± 112.2ms] and trial 8 [SCA6: 510.4 ± 112.9ms, control: 474.3 ± 115ms]. Significantly higher reaction times for SCA6 patients were observed for all trials, except for trial 1 [trial 1: mean difference (MD) = 19.1ms, Z = 2.49, *P* = 0.1; trial 2: MD = 29.4ms, Z = 4.34, *P* < 0.001; trial 3: MD = 38.5ms, Z = 5.64, *P* < 0.001; trial 4: MD = 41.1ms, Z = 5.56, *P* < 0.001; trial 5: MD = 56.3ms, Z = 7.62, *P* < 0.001; trial 6: MD = 52.5ms, Z = 7.3, *P* < 0.001; trial 7: MD = 47.2ms, Z = 6.2, *P* < 0.001; trial 8: MD = 36.1ms, Z = 4.78, *P* < 0.001; Wilcoxon rank sum test, with Bonferroni correction].

Next, we compared variances of pooled reaction times of correct button presses during training between groups and found a small but significant difference in variance, in which the SCA6 patient group showed a higher variance [SCA6: 1.258x10^4^ ms^2^, control: 1.172x10^4^ ms^2^, *F*_1,7107.19_ = 249.6, *P* < 0.001, Brown-Forsythe test of variances] (Fig 4E). We did the same analysis for pooled reaction times during the experimental sessions and found a consistent difference in variance between groups [SCA6: 1.342x10^4^ ms^2^, control: 1.163x10^4^ ms2, *F*_1,13277.38_ = 200.21, *P* < 0.001]. Together, these results indicate that average reaction times were significantly higher for SCA6 patients compared to controls. Furthermore, SCA6 patients also consistently showed a mildly reduced temporal precision, based on the observation that correct responses were more widely temporally distributed within this group.

### SCA6 patients show impaired learning of temporal interval order

We hypothesized that SCA6 patients would be impaired at learning sequences and temporal intervals of stimuli on the reactive timing task. In addition, controls could potentially use this type of learning as strategy to gain increased performance on conditions using fixed sequence or temporal intervals. To test this hypothesis, we visualized the AUCCR during FIX-FIX condition [SCA6: 0.152 ± 0.02, control: 0.231 ± 0.016], FIX-RANDOM condition [SCA6: 0.153 ± 0.02, control: 0.215 ± 0.017], RANDOM-FIX condition [SCA6: 0.134 ± 0.015, control: 0.181 ± 0.014], and RANDOM-RANDOM condition [SCA6: 0.138 ± 0.014, control: 0.193 ± 0.017] (Fig 4B). Using a repeated measures ANOVA we found a main effect of group, as well as a main effect of condition and an interaction effect [main effect of condition: *F*_1.71, 37.56_ = 22.82, *P* < 0.001; main effect of group: *F*_1,22_ = 7.1, *P* = 0.014; interaction condition x group: *F*_1.71,37.56_ = 4.12, *P* = 0.03, with Greenhouse-Geisser correction]. Closer inspection of only SCA6 performance furthermore showed a main effect of condition [*F*_1.56, 17.15_ = 4.29, *P* < 0.039, with Greenhouse-Geisser correction]. Planned contrasts comparing FIX-FIX condition with the other conditions revealed that the difference was located between the FIX-FIX condition and the RANDOM-FIX condition [*F*_1,11_ = 6.23, *P =* 0.03]. Performance of control participants also showed a significant main effect of condition [*F*_3,33_ = 23.45, *P* < 0.001] and contrasts indicated that AUCCR on the FIX-FIX condition was significantly higher compared to all other conditions [versus FIX-RANDOM: *F*_1,11_ = 15.49, *P* = 0.002; versus RANDOM-FIX: *F*_1,11_ = 72.05, *P* < 0.001; versus RANDOM-RANDOM: *F*_1,11_ = 21.02, *P* = 0.001] (Fig 4B). Together, these results show that although overall performance was reduced in the SCA6 patient group, randomizing the sequential order of stimuli resulted in reduced performance in both groups, suggesting that sequential order learning occurred in both groups. In contrast, randomizing the temporal interval order resulted in reduced performance only in the control group, but not in the SCA6 patient group. This suggests that only controls learned the temporal order of stimuli sufficiently, whereas this type of learning was reduced in SCA6 patients.

Complementary to the predictive timing task, we performed a PCA on AUCCR scores of all trial conditions to compare performance on groups independent of trial condition. Scores on all conditions were highly inter-correlated, at least *r* = 0.92 [*P <* 0.001, Pearson correlation]. Kaiser-Meyer-Olkin measure of sampling adequacy was 0.81, Bartlett’s test of sphericity was significant [χ^2^(6) = 173.12, *P* < 0.001] and all communalities were above 0.93, confirming that this data was suitable for PCA. We found that PC1 explained 96.2% of the variance and therefore we used this measure to represent performance on the experimental sessions of the reactive timing task. Confirming our earlier findings, PC1 differed significantly between groups [*t*_22_ = 2.69, *P* = 0.013, two-sample *t*-test].

### Performance on predictive and reactive motor timing tasks does not correlate with SARA scores

We hypothesized that if predictive and reactive motor timing functions are linked to cerebellar function, we could possibly find a relation between SCA6 symptom severity and performance on the tasks. To test this hypothesis, we performed an exploratory correlation analysis between performance on the predictive task and reactive timing task with individual scores on the Scale for the Assessment and Rating of Ataxia (SARA), which we obtained from 11 of the 12 SCA6 participants. Performance of both tasks was represented as the PC1 of scores on each task, as explained earlier. We did not find significant correlations between SARA score and scores on the predictive timing task (PC1 all correct responses) [*r* = -0.07, *P* = 0.85, Pearson correlation], PC1 of early correct responses [*r* = -0.509, *P* = 0.11, Pearson correlation], PC1 of late correct responses [*r* = 0.36, *P* = 0.27, Pearson correlation] and PC1 of reactive timing task [*r* = -0.5, *P* = 0.12, Pearson correlation].

## Discussion

In this study, we subjected cerebellar patients and control subjects to two finger-movement timing paradigms to find out to what extent the cerebellum contributes to spatio-temporal prediction and whether cerebellar dysfunction leads to changes in motor timing and coordination. We found that SCA6 patients were impaired at establishing spatio-temporal prediction of finger movements based on dynamic visual stimuli. Healthy control participants precisely timed their button-presses before a moving stimulus completely overlapped with a target stimulus, thereby anticipating or even ‘over-predicting’ their motor timing with approximately 66ms at the end of the training session, a behavioral effect that has also been observed previously [1]. Instead, cerebellar patients failed to establish spatio-temporal prediction and placed their button presses generally more temporally distributed, indicative of reduced temporal precision in responses. Concomitantly, SCA6 patients had significantly lower performance of early correct responses placed in the anticipatory period, but not of late correct responses placed in the period after the moving stimulus overlapped with the target stimulus.

Our results support previous work that has associated the cerebellum with spatio-temporal prediction processes and substantiate recent proposals arguing that the cerebellum is responsible for ‘monitoring’ ongoing timing and adjustment based on temporal predictions [32]. Striking similarities can be found with a study in which cerebellar patients and healthy participants had to precisely time their button press to intercept a moving target with a moving ball [2]. Lower hit-rates were observed in cerebellar patients and errors were equally distributed between (too) early and late responses. Interestingly, both this study and our results show a higher variability in patient response times, although in this study the variability was found to be limited to the late error trials, whereas we did not make such a distinction in our study. Using the same paradigm, cerebellar activations were found to be related to performance and were reduced in SCA patients [4]. Both these and our data indicate a predictive sub-second motor timing deficit in cerebellar patients, which could lead to impaired motor anticipation in daily life situations. For instance, Lang and Bastian (1999) showed that cerebellar patients failed to show anticipatory muscle activity when catching a falling weight, which made them unable to control the impact of the falling weight [33]. In a more general context, a considerable amount of literature has associated the cerebellum with temporal processing in the sub-second range, which are processes that are also engaged during predictive motor timing. A compelling example of this is that cerebellar patients show increased variation in timing, amplitude and velocity of finger opening in overarm throwing of a ball, which consequently results in reduced accuracy of throwing [34]. Moreover, cerebellar lesions in humans, as well as in rodents, affect the acquisition and particularly the timing of conditioned responses in Pavlovian eye blink conditioning [35], highlighting that the cerebellar cortex is specifically involved in the time course and amplitude of this learned motor behavior [36,37]. Other studies employing explicit timing tasks, such as rhythmic finger tapping and temporal interval discrimination and reproduction, have provided convincing evidence for cerebellar involvement in sub-second temporal processing. An increased temporal variability during rhythmic tapping with the finger or foot has been found in cerebellar patients [6,11], particularly associated with lesions in the lateral cerebellar regions [12]. Neuroimaging studies have found reliable activation of the cerebellum among several other areas in the cerebello-diencephalic-parietal network during finger-tapping in synchrony or continuation after tones at constant intervals [17–19]. Consistent with this finding, inactivating the ipsilateral cerebellar regions using repetitive transcranial magnetic stimulation (rTMS) introduced variability in the inter-tap interval [20]. Moreover, impaired temporal discrimination in the sub-second range has been found in both cerebellar patients and animal studies [6,13,14,38] (but see also: [39,40]). Gooch et al. (2010) however did not find impairments in temporal discrimination, although they did find impaired temporal estimation, production and reproduction in cerebellar lesion patients [41]. The reproduction of temporal intervals furthermore recruits cerebellar regions [15,16]. This cerebellar activation commonly coincides with activity in other cortical areas, *e*.*g*. basal ganglia, supplementary motor area (SMA), premotor area and inferior parietal cortex. Inactivating the left and right lateral cerebellum with rTMS impairs performance at a temporal reproduction task on short interval (millisecond range), but not at second range intervals [21]. The present view posits that the cerebellum is particularly involved in temporal processing at the sub-second scale, whereas the basal ganglia as part of a wider cortical network processes information at longer timescales [7,15,16,21,42–45]. This view is however still debated, as some researchers claim that the precise role of the cerebellum in timing processes has not been proven so far [46–48].

To assess the relationship between the performance of both tasks and indicators of SCA6 disease progression, we conducted a correlation analysis including the first PC as a measure of task performance in both predictive and reactive timing tasks and scores on the SARA [27]. Against expectations we failed to find a correlation between performance and SARA score. A possible explanation for this is that SARA score is based on multiple sub-scores assessing a wide range of functions, such as walking, stance, sitting and speech, whereas performance on our tasks depends specifically on the ability to precisely coordinate finger movements and to make precisely timed motor executions. A high SARA score does not necessarily translate into reduced fine motor skills, but could be the result of a broad spectrum of symptoms. Not finding a correlation could be explained by the fact that we had a rather small amount of participants for this exploratory correlation analysis, and that the SARA scores of our SCA6 participants were relatively low. Increasing the amount of participants with higher SARA scores could still reveal such an underlying relationship between task performance and SCA6 symptom severity.

In our reactive motor timing paradigm, we found that cerebellar patients overall scored worse than controls, which could indicate a general motor coordination deficit. Nevertheless, randomizing the sequence of stimuli resulted in a lower performance in both groups, indicating that sequence learning did occur in both SCA6 and control participants. Randomizing the temporal interval between markers, however, did not affect the performance of SCA6 patients, suggesting that temporal interval learning is impaired or that a deficit exists in the integration of spatial and temporal information in cerebellar patients. Learning of the sequence and/or temporal interval of stimuli could potentially aid in creating an expectation of which button to press and when to make the motor execution, respectively. This spatial and temporal expectation could be used within a strategy used during both the predictive and the reactive timing task, although randomization only resulted in performance differences during the reactive timing task. Learning of new motor sequences has been associated with enhanced activity in several brain areas, including the prefrontal cortex (PFC), putamen, intraparietal sulcus region (IPS), precuneus, premotor cortex (PMC), supplementary motor area (SMA) and cerebellum [49–57] (but see:[58]). It has been shown in multiple studies that during the initial stages of motor sequence learning frontal areas in combination with bilateral cerebellar regions show enhanced activation, after which the representation shifts to activation within the cortical-striatal circuit when the sequence has been learned [50,55–57]. Also, a shift in activity from the cerebellar cortex to deep cerebellar nuclei with sequence learning has been shown, suggesting segregation of neural networks involved at different stages of motor sequence learning [50]. Interestingly, the hippocampus has been shown to be active during learning of sequences [59], but also during retrieval of learned sequences [60]. A relationship between neural activity of hippocampal CA1 neurons and nonspatial sequence coding of odors has been found in rats, thus implying a role for the hippocampus in coding for sequential position of nonspatial objects [61]. These hippocampal related findings are interesting in the light of our recent observations of cerebellar-hippocampal interaction during spatio-temporal prediction [1], although the former sequences may not be related to timing at the millisecond level. Within the temporal domain, sustained activity in the lateral cerebellum and several structures within the neocortical-cerebellar network has been associated with temporal sequence learning in the form of intervals and rhythm [49,62,63]. Based on the cerebellar involvement in these forms of sequence learning, it seems likely that the observed deficits in temporal order learning can be attributed to cerebellar Purkinje cell specific degeneration in SCA6. These findings are in agreement with a previous study showing that cerebellar patients are indeed impaired on temporal, but also spatial sequence learning in a serial reaction time task [64]. Still, it should be noted that we were unable to detect sequence learning in cerebellar patients and healthy controls in our paradigm where spatio-temporal cues were available for motor prediction.

There are several limitations to our experimental approach. First, SCA6 covers a wide spectrum of symptoms [25,26], among others poor motor coordination of upper limbs, tremors, physical fatigue and oculomotor deficits. All of these may have affected participants’ ability to execute the tasks, since performance on both tasks was not only dependent on motor timing (*i*.*e*. pressing the button on time or as fast as possible), but also depended on precise finger movement coordination for pressing the right button. The possible influence of reduced motor coordination on predictive timing performance in SCA6 patients, as shown by reactive timing group differences, cannot be fully discounted. We attempted to minimize this influence by designing the paradigm in a way that movement of only one finger at a time was required instead of multiple fingers at the same time, and that the movement-speed of markers was acceptable for both SCA6 and control participants. As such, SCA6 patients showed increasing performance during the training on both tasks, which could indicate that reduced motor coordination has played a relatively limited role in our predictive task. We furthermore excluded SCA6 patients exhibiting unilateral or bilateral tremors or pronounced physical fatigue, thereby attempting to reduce these additional influences. Consequently, this reduced the average SARA score in our patient group. Our findings suggest however that cerebellar patients with relatively mild symptoms (*i*.*e*. having a low SARA score) already exhibit pronounced deficits in predictive motor timing functions. Attentional deficits may also have played a role in our study. Cerebellar patients have been shown to exhibit deficits in shifting attention between visual stimuli [65,66], although another study argues that response-related preparation processes rather than visuospatial attention shift are affected in cerebellar patients [67]. Both of these are potentially of importance, particularly during motor sequence learning, since differences in brain activation have been found between implicitly and explicitly learned motor sequences, suggesting that a different set of brain structures is activated depending on the degree of attentional resources assigned to the learned motor sequence [52]. Furthermore, cerebellar activity has been shown during a verbal working memory task [68] and a correlation between working memory and performance on temporal estimation, production and reproduction tasks has been found in subjects with cerebellar lesions [41], although it remains unclear to what extent SCA6 affects working memory in our patients. The potential influence of oculomotor deficits can also not be fully discounted. Future research using eye-tracking hardware may clarify to what extent eye movement deficits in SCA6 patients affect predictive motor timing. Recently it has been shown that in the mouse inducing the expression of C terminus polypeptides in Purkinje cells, as seen in SCA6 patients, yields both physiological and behavioral consequences, and may provide us with a new mouse model to study disease-related mechanisms of SCA6 in greater depth [23].

To conclude, we hereby provided evidence that SCA6 patients are impaired at establishing spatio-temporal prediction in timing of responses based on dynamic visual stimuli, reflecting a deficit in integration of spatial and temporal information and subsequently motor anticipation to upcoming events, using a task that demanded a prediction of when a stimulus would be at a certain target location. We propose that SCA6 patients are impaired in updating their forward internal model using spatial and temporal cues in the sub-second range, a process that has been postulated to require the cerebellum [69,70].
